# Infantile‐onset myoclonic developmental and epileptic encephalopathy: A new *RARS2* phenotype

**DOI:** 10.1002/epi4.12553

**Published:** 2021-11-18

**Authors:** Guillem de Valles‐Ibáñez, Michael S. Hildebrand, Melanie Bahlo, Chontelle King, Matthew Coleman, Timothy E. Green, John Goldsmith, Suzanne Davis, Deepak Gill, Simone Mandelstam, Ingrid E. Scheffer, Lynette G. Sadleir

**Affiliations:** ^1^ Department of Paediatrics and Child Health University of Otago Wellington New Zealand; ^2^ Department of Medicine Epilepsy Research Centre University of Melbourne Austin Health Melbourne Victoria Australia; ^3^ Murdoch Children’s Research Institute Royal Children’s Hospital Melbourne Victoria Australia; ^4^ Population Health and Immunity Division The Walter and Eliza Hall Institute of Medical Research Parkville Victoria Australia; ^5^ Department of Medical Biology University of Melbourne Parkville Victoria Australia; ^6^ Waikids Paediatric Service Waikato District Health Board Hamilton New Zealand; ^7^ Starship Children’s Hospital Auckland New Zealand; ^8^ T.Y. Nelson Department of Neurology and Neurosurgery The Children's Hospital at Westmead Sydney New South Wales Australia; ^9^ Department of Medical Imaging Royal Children’s Hospital Melbourne Victoria Australia; ^10^ The Florey Institute of Neuroscience and Mental Health Melbourne, Victoria Australia; ^11^ Departments of Paediatrics University of Melbourne Austin Health and Royal Children's Hospital Melbourne Victoria Australia

**Keywords:** developmental and epileptic encephalopathy, epilepsy, infantile, movement disorder, myoclonic, *RARS2*

## Abstract

Recessive variants in *RARS2*, a nuclear gene encoding a mitochondrial protein, were initially reported in pontocerebellar hypoplasia. Subsequently, a recessive *RARS2* early‐infantile (<12 weeks) developmental and epileptic encephalopathy was described with hypoglycaemia and lactic acidosis. Here, we describe two unrelated patients with a novel *RARS2* phenotype and reanalyse the published *RARS2* epilepsy phenotypes and variants. Our novel cases had infantile‐onset myoclonic developmental and epileptic encephalopathy, presenting with a progressive movement disorder from 9 months on a background of normal development. Development plateaued and regressed thereafter, with mild to profound impairment. Multiple drug‐resistant generalized and focal seizures occurred with episodes of non‐convulsive status epilepticus. Seizure types included absence, atonic, myoclonic, and focal seizures. Electroencephalograms showed diffuse slowing, multifocal, and generalised spike‐wave activity, activated by sleep. Both patients had compound heterozygous *RARS2* variants with likely impact on splicing and transcription. Remarkably, of the now 52 *RARS2* variants reported in 54 patients, our reanalysis found that 44 (85%) have been shown to or are predicted to affect splicing or gene expression leading to protein truncation or nonsense‐mediated decay. We expand the *RARS2* phenotypic spectrum to include infantile encephalopathy and suggest this gene is enriched for pathogenic variants that disrupt splicing.

## 1 INTRODUCTION

While *RARS2* was first identified as a recessive gene for pontocerebellar hypoplasia (PCH),^1^ a 2020 review of 25 cases with reported imaging found PCH in only 48%.^2^ There are now 52 published cases with *RARS2* variants with clinical information available for 43.^1^, ^2^, ^3^, ^4^, ^5^, ^6^, ^7^, ^8^, ^9^, ^10^, ^11^, ^12^, ^13^, ^14^, ^15^, ^16^, ^17^, ^18^, ^19^, ^20^, ^21^, ^22^, ^23^, ^24^, ^25^, ^26^, ^27^, ^28^, ^29^ Seizures are reported in 40 (93%) individuals with onset prior to age 3 months in 37 (93%). *RARS2* encodes a mitochondrial aminoacyl‐tRNA synthetase (mtARS) which catalyzes the attachment of arginine, vital for mtRNA–protein translation.^30^

**FIGURE 1:**
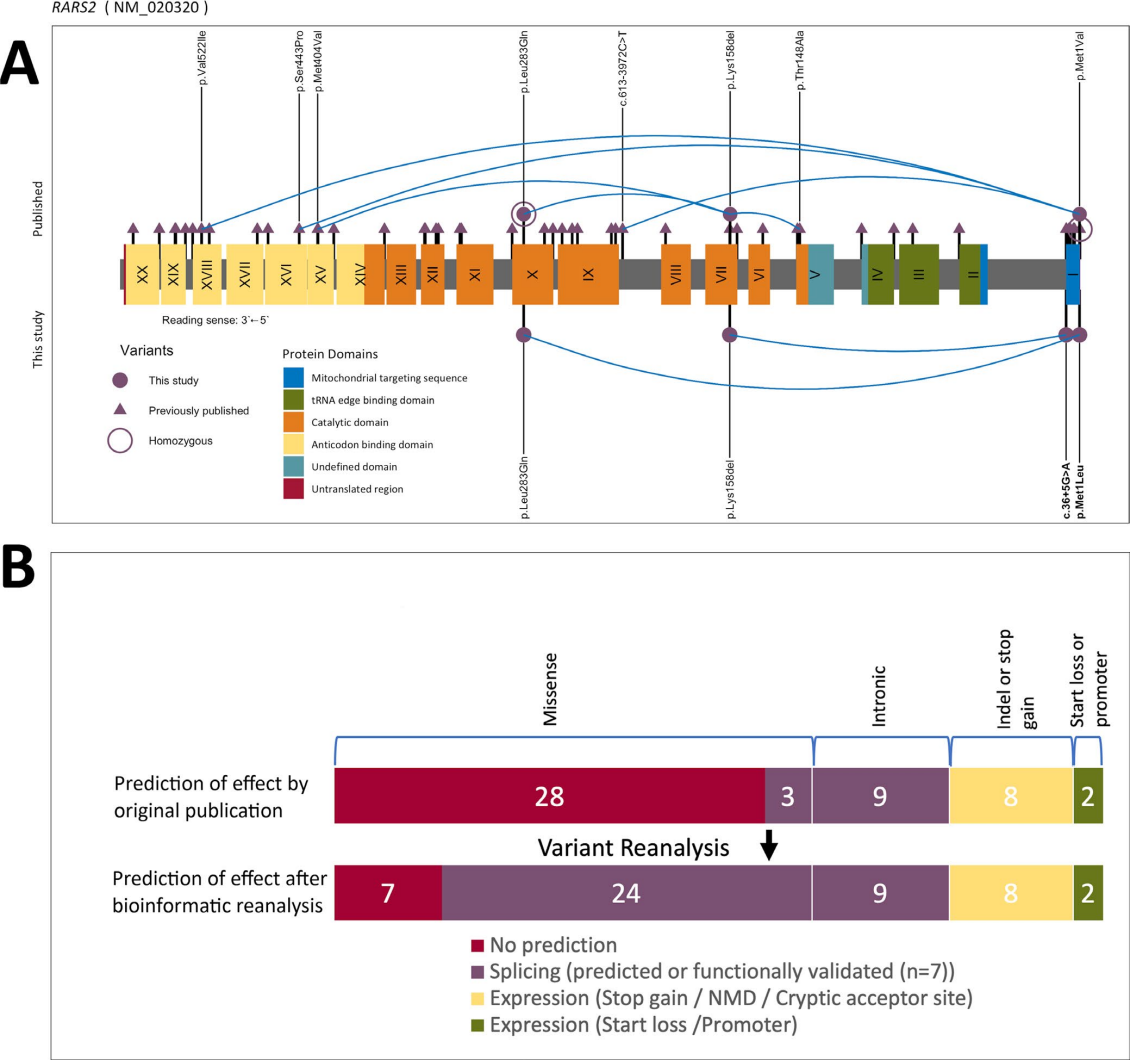
A, *RARS2* variants and their position in the *RARS2* gene. Previously published variants are shown above the protein. Previously published variants not found in our cases are represented with a triangle. Our cases are shown below the protein and variants found in our cases or affecting the same animoacid are represented by a circle. Blue lines link compound heterozygote variants identified in our cases and published cases who have at least one variant also found in our cases. cDNA changes in bold represent novel variants found in this study. Introns and UTRs are represented in a 1:100 scale. B, Effect on expression and splicing of the published *RARS2* variants. The top bar represents represent the effect of the variants as considered by original publication and the bottom bar represents the effect of the same variants after our bioinformatic reanalysis. NMD, nonsense‐mediated decay

The reported 50 pathogenic *RARS2* variants (Table S1) occur in families as compound heterozygous mutations in 81% and are most commonly missense (62%. 31/50) (Figure 1B). There are ten recurrent variant positions (p.Met1(Val/Leu)^15^, ^18^, ^29^; p.Gln12Arg^4^, ^6^, ^13^, ^28^; c.110+5A>G^1^, ^8^, ^13^; p.Lys158del^3^, ^7^, ^28^; p.Gln208*^20^, ^26^; p.Arg258His^5^, ^26^; p.Leu283Gln^7^, ^23^, ^25^; p.Met342Ile^12^, ^20^; p.Asp515Gly^9^, ^22^; p.Val522Ile^28^, ^29^); however no clear genotype–phenotype correlation has been identified.^2^

We describe a new *RARS2* phenotype of infantile‐onset myoclonic developmental and epileptic encephalopathy in two unrelated children and compare this to the epileptology in the previously reported cases. We reanalyze the published variants and suggest there is enrichment for variants that disrupt splicing in this gene.

## 2 METHODS

Two children had compound heterozygous *RARS2* (RefSeq: NM_020320.5) pathogenic variants identified. Trio genome sequencing was performed on Case A and her unaffected parents as part of our epilepsy genetics research cohort of 250 patients with developmental and epileptic encephalopathies (DEEs) who underwent whole genome sequencing (28) or whole exome sequencing (222) analysis. Case B had singleton whole exome sequencing. Variants were called using the standard genome analysis toolkit pipeline and annotated with SnpEff. We filtered coding variants with a frequency <0.0001 in population databases (1000 Genomes phase 3, NHLBI GO Exome Sequencing Project, ExAC, and gnomAD) and an impact severity predicted by SnpEff of ‘HIGH’ or ‘MEDIUM’. Variants were validated and segregation performed with Sanger sequencing. We used Human Splicing Finder (HSF), SpliceAI, and Transcript Inferred Pathogenicity Score (TraP) to predict impact on splicing by our variants and published variants (Table S1).

We reviewed epilepsy and medical history, examination, magnetic resonance imaging (MRI), and electroencephalogram (EEG) findings. The study was approved by Austin Health and New Zealand Health and Disability Ethics Committees. Written informed consent was obtained from the parents.

We reviewed *RARS2* publications prior to Aug 2021 and identified 52 novel *RARS2* cases (Table S1).^1^, ^2^, ^3^, ^4^, ^5^, ^6^, ^7^, ^8^, ^9^, ^10^, ^11^, ^12^, ^13^, ^14^, ^15^, ^16^, ^17^, ^18^, ^19^, ^20^, ^21^, ^22^, ^23^, ^24^, ^25^, ^26^, ^27^, ^28^, ^29^ We compared our cases with the epileptology reported for *RARS2* encephalopathy; adequate information was only available for 15/52 reported cases (Table 1).^2^, ^3^, ^4^, ^5^, ^6^, ^7^, ^8^, ^9^, ^10^, ^11^

**TABLE 1:** Symptoms that child presented with are shaded yellow

CASE	CASE A	CASE B	Case 1	Case 2	Case 3	Case 4	Case 5	Case 6	Case 7
Publication	Ours	Ours	Glamuzina et al, 2012	Cassandrini et al, 2013	Cassandrini et al, 2013	Cassandrini et al, 2013	Kastrissianikis et al, 2013	Kastrissianikis et al, 2013	Rankin et al, 2010
Patient (sex, age at study)	A (F,6 y ‐died)	B (F, 10 y)	IV‐1.1 (F, 2 y)	A‐01 (M,11 y)	B‐01 (F, 9 y)	B‐02 (F, 3 y)	Sibling 1 (F, 3 y)	Sibling 2 (M, 12 m)	1 (F, 4 y 6 m)
Epilepsy Syndrome	Infantile onset DEE	Infantile onset DEE	Neonatal onset DEE	Neonatal onset DEE	Neonatal onset DEE	Neonatal onset DEE	Neonatal onset DEE	Neonatal onset DEE	Neonatal onset DEE
Seizure onset	2 y 2 m	3 y	4 w	11 d	20 d	11 d	2 w	1 d	2 d
First seizure type	My; A	A	Motor SE	FM (clonic)	Motor SE	FM (clonic)	TC	FM (clonic)	My
Subsequent seizures	FBTC; T; Ab (atypical); Non‐convulsive & motor SE	Ab (atypical); MyA; non‐convulsive SE	UK; TC	FM (multifocal)	FM (multifocal)	FM (multifocal)	TC	FM (multifocal)	My
Pharmaco‐resistant	Y	Y	Y	Y	Y	Y	Y	Y	NR
Developmental concern: onset age: outcome	17 m: Profound ID (G‐tube)	15 m: Mild ID	Birth: Profound DD (G‐tube)	Birth: Severe ID	Birth: Severe ID (G‐tube)	Unclear: Severe DD	Unclear <3 m: Severe DD (G‐tube)	Birth: Severe DD	Birth: Profound DD (G‐tube)
Regression (age)	Y (17 m)	Y (5 y)	N	N	N	N	N	N	N
Examination (neuro)	Central hypotonia, increased tone, reflexes	Ataxia, dysarthria, café au lait lesions, freckling	Hypotonia	Initial hypotonia developed spastic quadriplegia	Initial hypotonia developed spastic quadriplegia	Spastic quadriplegia	Spastic quadriplegia	Increased tone & reflexes	Hypotonia & increased reflexes
Microcephaly	N	N	Y	Y	Y	Y	Y	N	Y
Movement disorder: age of onset & type	Yes: 9 m, myoclonus	Yes: 8 m, myoclonus	Yes: 1 m, severe dystonia	Possibly	Possibly	Possibly	N	N	N
EEG	2 y 4 m: MFD; 2 y 5 m & 2 y 9 m: Slow, MFD; 3 y 4 m & 4 y 5 m: Slow, MFD, GSW, PSW	2 y: Normal; 3 y: GSW; 7 y 9 m: Slow, GSW, 8 y & 10 y: Slow, GSW, MFD, PPR	4 w: BS; 6 m: Slow, bifrontal discharges	Slow, MFD	Slow, MFD	Slow, MFD	14 w: Focal slow, MFD; 16 m: CSWS	1 d: Normal; 3 w: BS in sleep, MFD	1 w: Discharges, 2 y: GSW
MRI	Subtly small pons & cerebellum, mild PVWM hyper‐intensity	Mild cortical atrophy; Focal lesion right cerebellum	Pontocerebellar hypoplasia; cortical & optic nerves atrophy	Cerebellar vermis hypoplasia; Cortical & cerebellar atrophy	Progressive cortical & pontocerebellar atrophy	Progressive cortical & pontocerebellar atrophy	Cortical & cerebellar atrophy	Cortical & cerebellar atrophy	Cortical & pontocerebellar atrophy
Metabolic abnormalities (peak blood lactate level)^a^	Normal (blood 1.3 to 2.2 mmol/L; CSF 1.4 mmol/L)	Normal (blood 2.0 mmol/L; CSF 1.4mmol/L)	Neonatal hypoglycaemia & lactic acidosis (14.2 mmol/L)	Neonatal lactic acidosis (6.7 mmol/L)	Neonatal hypoglycaemia & lactic acidosis (3.7 mmol/L)	Neonatal onset of lactic acidosis (2.4 mmol/L)	Mildly elevated CSF lactate (blood lactate normal)	Neonatal hypoglycaemia & lactatic acidosis (3.7 mmol/L)	Neonatal hypoglycaemia & lactic acidosis (16 mmol/L)
Variant 1*	c.848T>A; p.L283Q^p^	c.36+5G>A^m^;intronic ‐	c.1211T>A; p.M404K^m^	c.25G>A; p.I9V^p^	c.734G>A; p.R245Q^m^	c.734G>A; p.R245Q^m^	c.773G>A; p.R258H	c.773G>A; p.R258H	c.1024A>G; p.M342V^m^
SIFT	D (0)	‐	D (0)	T (0.12)	D (0)	D (0)	D (0)	D (0)	D (0)
PolyPhen	B (0.021)	10.96	D (0.953)	B (0.03)	D (0.997)	D (0.997)	D (0.992)	D (0.992)	P (0.688)
CADD	22	‐4.3	29.5	15.42	32	32	34	34	24.7
GERP RS	3.97	0	5.9699	3.868	6.17	6.17	5.51	5.51	4.659
gnomADex	2	0	0	5	6	6	70	70	0
gnomADgen	0		0	0	0	0	14	14	0
Variant 2*	c.1A>T; p.M1L ^m^	c.472_474delAAA;p.K158delK^p^ ‐	c.471_473delCAA;p.K158delK^p^ ‐	c.1586+3A>T^m^ ‐	c.1406G>A; p.R469H^p^	c.1406G>A; p.R469H^p^	c.1651‐2A>G ‐	c.1651‐2A>G ‐	c.35A>G; p.Q12R^p^
SIFT	D (0)	‐	‐	‐	D (0)	D (0)	‐	‐	T (0.07)
PolyPhen	B (0.0998)	‐	‐	23	D (1)	D (1)	33	33	B (0)
CADD	27	4.4327	4.84	5.28	32	32	5.1999	5.1999	23.3
GERP RS	5.5	32	0	0	5.11	5.11	0	0	3.97
gnomADex	2	3	0	0	0	0	0	0	17
gnomADgen	0				0	0			0

CASE	Case 8	Case 9	Case 10	Case 11	Case 12	Case 13	Case 14	Case 15
Publication	Nevanlinna et al, 2020	Ngoh et al, 2016	Ngoh et al, 2016	Nishri et al, 2016	Nishri et al, 2016	Van Dijk et al, 2017	Zhang et al, 2018	Xu et al, 2020
Patient (sex, age at study)	1 (M, 11 y‐died)	Patient 1 (M, 11 y)	Patient 2 (M, 6 y)	II‐3 (F, 3 y‐died)	II‐4 (M, 4 y‐died)	B (F, 3 m‐died)	I (M, 3 y)	(F, 7 m)
Epilepsy Syndrome	Neonatal onset DEE	Neonatal onset DEE to Infantile spasms	Early onset DEE to infantile spasms	Early onset DEE	Early onset DEE	Early onset DEE	Early onset DEE	Neonatal onset DEE to Infantile Spasms
Seizure onset	6 w	5 w	8 w	9 w	12 w	Unclear <12 w	12 w	19 d
First seizure type	FTBCS	Motor seizures (clonic)	Motor seizures (clonic)	FM (clonic)	FM (clonic)	FM (clonic)	FM (multifocal)	My
Subsequent seizures	FM; FIAS; My: non‐convulsive SE	ES; T; GTCS (+SE)	ES; My; ES	FM (multifocal), My	My; T	Motor SE	FM (multifocal)	Convulsive status epilepticus, ES
Pharmaco‐resistant	Y	Y	Y	Y	Y	Y	Y	Y
Developmental concern: onset age: outcome	2 m: Profound ID (G‐tube)	1 m: Profound ID (G‐tube)	2 m: Severe DD (G‐tube)	Birth: Profound DD (G‐tube)	Unclear <2 m: Profound DD	Unclear<3 m: Severe DD	5 m: Profound DD (G‐Tube)	Unclear: Severe DD
Regression (age)	N	N	N	Y (4m)	Y (9m)	N	N	NR
Examination (neuro)	Initial hypotonia developed spastic quadriplegia	Spastic quadriplegia	Axial hypotonia & spastic quadriplegia	Increased reflexes	Axial hypotonia & spastic quadriplegia	Hypotonia	Initial hypotonia, later hypertonia	NR
Microcephaly	Y	Y	Y	Y	N	NR	Y	NR
Movement disorder: age of onset & type	N	N	N	N	N	N	N	NR
EEG	3 m: Normal; 4 m: Slow, MFD	6 w: Normal; 8 m: modified Hyps; 11 m: MFD	5 m: modified Hyps; 2.5 y: MFD	9 w: Normal; 4 m: Slow, FD, PSW, EDE; 3 y: MFD (migratory)	3 m: FD; 9 m: MFD, PSW	3 m: MFD	6 m: Slow, MFD	24 d: BS
MRI	Cortical & basal ganglia atrophy	Thin corpus callosum, small cerebellum	Thin corpus callosum, small cerebellum	Cortical & cerebellar atrophy	Cortical atrophy	Cortical atrophy	Cortical, basal ganglia & white matter atrophy	Cortical atrophy, hypomylenation
Metabolic abnormalities (peak blood lactate level)^a^	Elevated lactate (5.3 mmol/L)	Neonatal hypoglycaemia; elevated lactate (4.9 mmol/L)	6 y‐mild elevation of CSF glycine (blood lactate normal)	Normal	Normal	MRS lactate peak (CSF & blood normal)	4m: Lactic acidosis (9.5 mmol/L)	Neonatal Hypoglycaemia & lactic acidosis (value NR)
Variant 1*	c.795delA; p.E265Dfs^m^	c.848T>A; p.L283Q	c.848T>A; p.L283Q	c.110+5A>G^m^; intronic ‐	c.110+5A>G^m^;intronic ‐	c.1544A>G; p.D515G^m^	c.1718C>T; p.T573I	c.282_285delAGAG^p^ ‐
SIFT	‐	‐	‐	‐	‐	D (0.01)	T (0.06)	‐
PolyPhen	‐	‐	‐	16.17	16.17	D (0.901)	B (0.411)	‐
CADD	‐	‐	‐	5.4499	5.4499	25.8	23.2	‐
GERP RS	4.2	4.84	4.84	3	3	5.3699	4.65	6 (0)
gnomADex	0	2	2	0	0	7	26	1 (0)
gnomADgen	0	0	0			1	0	
Variant 2*	c.961C>T; p.L321F^p^	c.472_474delAAA;p.K158delK D (0)	c.472_474delAAA;p.K158delK D (0)	c.878+5G>T^p^ ‐	c.878+5G>T^p^ ‐	c.297+2T>G^p^ ‐	c.991A>G; p.I331V	c.773G>A p.R258H^m^
SIFT	D (0.03)	D (0.998)	D (0.998)	‐	‐	‐	T (0.42)	0 (D)
PolyPhen	D (0.874)	27	27	18.87	18.87	‐	B (0.291)	0.992 (D)
CADD	31	5.5	5.5	5.51	5.51	4.29	22.1	33
GERP RS	5.599	0	0	0	0	0	5.8299	5.51
gnomADex	1	0	0	0	0	0	8831 + 300	70 (0)
gnomADgen	0						1071 + 35	14 (0)

Abbreviations: A, atonic seizure; Ab, absence seizure; B, Benign; BS, burst suppression; CSF, cerebrospinal fluid; CSWS, continuous spike and wave in sleep; D, Damaging; d, days; DD, developmental delay; DEE, developmental and epileptic encephalopathy; EDE, electro decremental event; ES, epileptic spasm; F, female; FBTC, focal to bilateral tonic clonic seizure; FIAS, focal impaired awareness; FM, focal motor seizure; GSW, generalized spike and slow wave; GTCS, generalized tonic clonic seizure; Hyps, hypsarrhythmia; ID, intellectual disability; M, male; ^m^, maternal inheritance; m, months; MFD, multifocal discharges; MRS, magnetic resonance spectroscopy; My, myoclonic seizure; MyA, myoclonic atonic seizure; N, no; NR, not recorded; ^p^, paternal inheritance; P, Probably damaging; PPR, photo paroxysmal response; PSW, polyspike and slow wave; PVWM, periventricular white matter; SE, status epilepticus; seizure; T, Tolerated; T, tonic seizure; TC, tonic clonic seizure; UK, unknown seizure type; w, weeks; y, years; Y, yes.

^a^lactate levels are in blood unless otherwise specified.

*In‐silico predictions, GERP conservation values and genetic database frequencies obtained from: CADD, SIFT, PolyPhen, GERP: https://cadd.gs.washington.edu/snv, gnomAD: https://gnomad.broadinstitute.org/gene/ENSG00000146282?dataset=gnomad_r2_1

## 3 RESULTS

### 3.1 Clinical phenotyping

Case A, who died at 6 years, was born following a normal pregnancy to unrelated parents of European ancestry. Early development was normal. At 9 months, she developed action myoclonus of her hands which became more prominent over time. By 17 months, she was ataxic and had dysphagia, with developmental regression. At 2 years 2 months, she developed myoclonic and atonic seizures associated with fever. By 2.5 years, she could no longer sit or speak and required gastrostomy‐tube feeding. She had central hypotonia with increased peripheral tone, increased reflexes, and dystonia. She had continuous multifocal myoclonus of her limbs and face. Electroencephalogram showed that her myoclonus did not correlate with epileptiform discharges, despite frequent multifocal and generalised spike wave (GSW) and polyspike wave, together with diffuse background slowing. The GSW was maximal independently in the bifrontal and bioccipital areas and continuous in sleep. She developed focal motor seizures at 2.5 years. Tonic, atypical absence, and definite epileptic myoclonic seizures were apparent by 3 years. By 3.5 years, she had convulsive and non‐convulsive status epilepticus. Her epilepsy was drug‐resistant; oral prednisolone and diazepam resulted in short‐term improvement of her myoclonus and EEG. She was noted to have accelerated growth without pubertal signs from 4 years. At 6 years, she died following convulsive status epilepticus.

Magnetic resonance imaging of the brain showed a thick rostrum/genu of the corpus callosum, mild periventricular white matter hyperintensity, and structurally normal but the small pons and cerebellum (just under the 3rd percentile volume for age). Metabolic investigations including blood lactate, skin, rectal, and muscle biopsies were uninformative.

Case B is a 10‐year‐old girl born to an unrelated Japanese mother and Australian father. She developed myoclonus of her fingers and hands at 8 months; an EEG capturing myoclonus at age 2 years was normal. The myoclonus continued, and at 3 years she developed atonic seizures. At 4 years of age, her myoclonus became more prominent and atypical absence seizures began. She developed episodes of non‐convulsive status epilepticus from 6.5 years that were responsive to steroids. At 8 years, she remains drug‐resistant with daily myoclonic, atypical absence and atonic seizures and the movement disorder (Video S1). She has had over 10 anti‐seizure medicines and the ketogenic diet.

Early development was normal with single words at 1 year and walking at 14 months; however, by 15 months her development slowed, and she remained ataxic. Cognitive development plateaued at 4 years, and her speech regressed from 5 years. By 8 years, her speech was limited to 15‐word sentences in English and Japanese (Video S2).

On examination, she had 30 café‐au‐lait lesions and axillary freckling. She was ataxic and dysarthric and had prominent widespread myoclonus but was otherwise neurologically normal with normal head circumference. Her EEG showed diffuse slowing and very frequent multifocal spikes and 5 seconds bursts of GSW. The GSW was either maximal bifrontal or bioccipital, and she developed photosensitivity by 7 years. In sleep, bursts of GSW evolved to long runs of rhythmic monomorphic high voltage delta lasting up to 30 minutes. Video‐EEG monitoring at 8 years captured atypical absence seizures with eyelid fluttering and gradual loss of tone with paroxysms of GSW. Magnetic resonance imaging shows generalized sulcal prominence and a non‐specific focal lesion in the right cerebellar white matter. Lactate and metabolic markers were unremarkable.

The *RARS2* literature included 15 cases with sufficient clinical information to compare with our patients (Table 1). All had drug‐resistant developmental and epileptic encephalopathies. Eight had neonatal onset, and seven had onset from 4 weeks to 3 months. They presented with seizures (13/15), developmental delay (10/15), hypoglycemia (6/15), and lactic acidosis (8/15). All showed delay in development by 5 months and seven in the neonatal period. Five children died aged 3 months to 11 years, and surviving children had severe to profound disability.

### 3.2 Molecular findings

Our two children had compound heterozygous pathogenic variants in *RARS2* (Table 1).

For Case A, the maternally inherited start‐loss variant is at the same position as previously published variants,^15^, ^18^, ^29^ and the paternally inherited missense variant is a recurrent pathogenic variant^7^, ^23^, ^25^ (Table 1). No other plausible pathogenic variants were identified in the trio genome.

For Case B, the paternally inherited in‐frame deletion is a recurrent pathogenic variant reported in three patients.^3^, ^7^, ^28^ The novel maternally inherited splice site variant is predicted by three independent in silico splicing prediction tools (HSF score 90.21 > 80.64 [−10.61%] [alteration of the wild‐type donor site, the most probably affecting splicing], TraP score 0.856 [probably damaging], and SpliceAI score 0.1593 for donor loss [low probability]) to lead to loss of the wild type donor site and disrupt splicing. This patient also has a mosaic pathogenic stop‐gain variant in *NF1* (Table S2). Given that only 3.7% of children with *NF1* have epilepsy,^31^ that *NF1* epilepsy is usually focal,^31^ and that the six cases with her *NF1* variant did not have seizures,^32^, ^33^, ^34^ this mosaic variant is unlikely to be contributing to her epilepsy phenotype.

Reanalysis of the published missense variants predicts that 24/31 (77%) affect splicing, (Figure 1B, Table S1) meaning that 86% (43/50) of all variants likely affect expression.

## 4 DISCUSSION

Here we describe two children with a new *RARS2* phenotype that is quite distinct from the previously described early infantile developmental and epileptic encephalopathy *RARS2* phenotype. In the 15 published cases that describe the *RARS2* epilepsy phenotype in detail,^2^, ^3^, ^4^, ^5^, ^6^, ^7^, ^8^, ^9^, ^10^, ^11^, ^12^ seizure onset occurred by 3 months in the setting of abnormal development. In contrast, our children presented with a progressive movement disorder at 8 and 9 months on a background of normal development. They subsequently developed a myoclonic developmental and epileptic encephalopathy with developmental slowing between 15 and 17 months and seizure onset after 2 years of age.

Of the previously published cases in Table 1, only Case 1 was recognised to have a movement disorder which was described as near continuous jerks and severe dystonia from 4 weeks.^3^ The distinction between a non‐epileptic and epileptic myoclonus can be challenging. Interestingly Case 2, 3, and 4 were described as having frequent upper‐limb myoclonus after the first year of life which may have been an unrecognised movement disorder.^4^

Eight families share at least one of the variants or a variant affecting the same amino acid as the variants in our cases (Figure 1A); for six families comparable clinical information is provided.^3^, ^7^, ^15^, ^18^, ^23^, ^25^ Four had early infantile‐onset DEE.^3^, ^7^, ^15^, ^18^ The other two families with three affected individuals had homozygous p.Leu283Gln variants.^23^, ^25^ Although there was limited epilepsy phenotyping information, two were siblings described as having progressive myoclonic epilepsy with myoclonic jerks noted on day one, childhood febrile seizures, and mild to severe intellectual disability.^23^ The third had adolescent onset cerebellar ataxia, a single seizure, and delayed development.^25^ In addition to the six families with shared variants, there is one family with a homozygous promotor variant (c.‐2A>G variant) likely to have a similar effect to the start‐loss variant in our Case A. This family had two children with slow development, seizure onset at 9 months in one, and developmental regression without the movement disorder.^16^

*RARS2* is highly tolerant to missense variation (Z = −0.06, gnomAD) which, although not unusual for recessive genes, is out of keeping with the proportion (62%) of pathogenic missense variants reported in this gene. As most of these exonic *RARS2* variants occur close to exon boundaries (Figure 1A), we theorised that they may affect splicing. Indeed, our bioinformatic reanalysis predicted that a high proportion (24/31, 77%) of these *RARS2* missense variants are predicted to affect expression. Although these predictions are based only on bioinformatic tools, aberrant splicing of *RARS2* has been functionally confirmed in the recurrent p.Gln12Arg variant using an exon trap vector in vitro.^6^

Overall, our bioinformatic reanalysis suggests that 85% of the now 52 *RARS2* variants likely impact on splicing or expression of the gene and that most cases with *RARS2* encephalopathy have at least one variant with this effect (46/54 with two variants, 7/54 with one variant) (Table S1). Mitochondrial genes, such as *RARS2*, are required for adenosine triphosphate production^4^, ^30^ and so are essential for organs with high energy demand, such as the brain. Abnormal splicing in these genes is particularly problematic in the brain as mitochondrial human aminoacyl‐tRNA synthetases already have low splicing efficiency in neurons compared to other tissues due to naturally occurring weak splice sites.^35^ We speculate that in *RARS2*, these low levels do not reach the threshold for sufficient mitochondrial function and result in the progressive neurological disease.^1^, ^30^, ^35^

## CONFLICT OF INTERESTS

Prof Sadleir is funded by the Health Research Council of New Zealand and Cure Kids New Zealand. She is a consultant for the Epilepsy Consortium and has received travel grants from Seqirus and Nutricia. She has received research grants from Zynerba. Prof Scheffer serves/has served on scientific advisory boards/consulted for UCB, Eisai, GlaxoSmithKline, BioMarin, Nutricia, Rogcon, Xenon Pharmaceuticals, Zynerba Pharmaceuticals, Ovid Therapeutics, Atheneum Partners, Chiesi, Encoded Therapeutics and Knopp Biosciences; has received speaker honoraria from GlaxoSmithKline, UCB, BioMarin, Biocodex and Eisai; has received funding for travel from UCB, Biocodex, GlaxoSmithKline, Biomarin and Eisai; and has served as an investigator for Zogenix, Zynerba, Ultragenyx, GW Pharma, UCB, Eisai, Anavex Life Sciences, Ovid Therapeutics, Epigenyx, Encoded Therapeutics and Marinus. She may accrue future revenue on pending patent WO2009/086591; has a patent for SCN1A testing held by Bionomics Inc and licensed to various diagnostic companies; and has a patent molecular diagnostic/ therapeutic target for benign familial infantile epilepsy (BFIE) [PRRT2] WO/2013/059884 with royalties paid. The remaining authors have no conflicts of interest.

## ETHICAL APPROVAL

The authors confirm that we have read the Journal's position on issues involved in ethical publication and affirm that this report is consistent with those guidelines.

## Supporting information

Table S1

Table S2

Video S1

Video S2
