# Supplementary material for: Infantile‐onset myoclonic developmental and epileptic encephalopathy: A new RARS2 phenotype
Source: Epilepsia Open. 2021 Nov 18;7(1):170–80. doi: 10.1002/epi4.12553 (PMC8886097; doi:10.1002/epi4.12553)
Supplement: Supplementary file 1 — Table S1 [file EPI4-7-170-s004.docx]

| **Patient** | **Publication** | **Variants** | **Effect on  splicing/expression  as per previous publication** | **Splicing predictions as per bioinformatic algorithms** | | | **Effect on  spicing/expression  as per reanalysis** |
| --- | --- | --- | --- | --- | --- | --- | --- |
|  |  |  |  | TRAP score^1^ | Splice AI^2^ AG/AL/DG/DL | Human Splicing Finder^3^ |  |
| **Case A** | This study | c.1A>T;p.M1L* | - | 0 | 0/0/0/0 | No alteration | YES |
|  |  | c.848T>A;p.L283Q | - | 0.436 | 0/0/0.0001/0.1564 | ESE alteration, cryptic acceptor:  56.96>84.83 (48.93%) | YES |
| **Case B** | This study | c.36+5G>A | - | 0.856 | 0.0001/0/0.0035/0.1593 | WT donor:  90.21>80.64 (-10.61%) | YES |
|  |  | c.472_474delAAA;p.K158delK | - | N/A | N/A | ESE alteration, cryptic acceptor:  58.16>75.4 (29.64%) | YES |
| **Case 1** | Glamuzina et al, 2012^4^ | c.1211T>A;p.M404K | Not provided | 0.595 | 0/0/0.0008/0.0028 | Cryptic acceptor: 38.84>66.71 (71.76%), ESE alteration | YES |
|  |  | c.471_473delCAA;p.K158delK | Not provided | N/A | N/A | ESS creation, ESE alteration | YES |
| **Case 2** | Cassandrini et al, 2013^5^ | c.25G>A;p.I9V | Not provided | 0.138 | 0.0002/0/0/0.0072 | ESS creation | YES |
|  |  | c.1586+3A>T | Functionally validated: splicing mutation deletes 25 amino acids in the anticodon binding domain, shortened but functional version of the protein^5^ | 0.968 | 0.0161/0/0.0079/0.9258 | WT donor: 82.43>69.55 (-15.63%), MaxEnt donor: 8.4>3.87 (-53.93%) | YES |
| **Case 3** | Cassandrini et al, 2013^5^ | c.734G>A;p.R245Q | Not provided | 0.662 | 0.0002/0/0.0005/0.0003 | Cryptic acceptor, ESS creation | YES |
|  |  | c.1406G>A;p.R469H | Not provided | 0.044 | 0.0001/0/0/0.0005 | ESE alteration | YES |
| **Case 4** | Cassandrini et al, 2013^5^ | c.734G>A;p.R245Q | Not provided | 0.662 | 0.0002/0/0.0005/0.0003 | Cryptic acceptor, ESS creation | YES |
|  |  | c.1406G>A;p.R469H | Not provided | 0.044 | 0.0001/0/0/0.0005 | ESE alteration | YES |
| **Case 5** | Kastrissianikis et al, 2013^6^ | c.773G>A;p.R258H | Not provided | 0.138 | 0.0003/0.0029/0/0 | ESE alteration | YES |
|  |  | c.1651-2A>G | Not provided | 0.559 | 0.0049/0.9794/0/0 | WT acceptor: 90.62>62.75 (-30.75%), MaxEnt acceptor: 9.26>1.3 (-85.96%) | YES |
| **Case 6** | Kastrissianikis et al, 2013^6^ | c.773G>A;p.R258H | Not provided | 0.138 | 0.0003/0.0029/0/0 | ESE alteration | YES |
|  |  | c.1651-2A>G | Not provided | 0.559 | 0.0049/0.9794/0/0 | WT acceptor: 90.62>62.75 (-30.75%), MaxEnt acceptor: 9.26>1.3 (-85.96%) | YES |
| **Case 7** | Rankin et al, 2010^7^ | c.1024A>G;p.M342V | Not provided | 0.303 | 0.0022/0/0/0 | Cryptic acceptor: 41.24>68.39 (65.83%), ESS creation | YES |
|  |  | c.35A>G;p.Q12R | Functionally validated: insertion of an intron with a premature stop codon^7^ | 0.968 | 0/0/0.003/0.5824 | ESS creation, ESE alteration | YES |
| **Case 8** | Nevanlinna et al, 2020^8^ | c.795delA;p.E265Dfs*16 | Functionally validated: frameshift variant forces transcript to NMD^8^ | N/A | N/A | ESE alteration, cryptic donor:  28.71>65.94 (129.68%). | YES |
|  |  | c.961C>T;p.L321F | Not provided | 0.061 | 0/0/0/0.1109 | ESS creation | YES |
| **Case 9** | Ngoh et al, 2016^9^ | c.472_474del;p.K158delK | Not provided | N/A | N/A | ESS creation | YES |
|  |  | c.848T>A;p.L283Q | Not provided | 0.436 | 0/0/0.0001/0.1564 | ESE alteration, cryptic acceptor:  56.96>84.83 (48.93%) | YES |
| **Case 10** | Ngoh et al, 2016^9^ | c.472_474del;p.K158delK | Not provided | N/A | N/A | ESS creation | YES |
|  |  | c.848T>A;p.L283Q | Not provided | 0.436 | 0/0/0.0001/0.1564 | ESE alteration, cryptic acceptor:  56.96>84.83 (48.93%) | YES |
| **Case 11** | Nishri et al, 2016^10^ | c.110+5A>G | Not provided | 0.716 | 0/0/0.5162/0.7199 | Cryptic donor | YES |
|  |  | c.878+5G>T | Not provided | 0.932 | 0/0.0003/0.0041/0.6211 | WT donor:  79.48>69.78 (-12.2%), MaxEnt acceptor:  2.83>4.23 (49.47%) | YES |
| **Case 12** | Nishri et al, 2016^10^ | c.110+5A>G | Not provided | 0.716 | 0/0/0.5162/0.7199 | Cryptic donor | YES |
|  |  | c.878+5G>T | Not provided | 0.932 | 0/0.0003/0.0041/0.6211 | WT donor | YES |
| **Case 13** | Van Dijk et al, 2017^11^ | c.1544A>G;p.D515G | Splicing effect considered based on location^11^ | 0.084 | 0.0012/0.1197/0.0007/0 | ESE alteration | YES |
|  |  | c.297+2T>G | Functionally validated: skipping of exon 4^11^ | 0.964 | 0/0.0001/0/0.9832 | HSF WT donor: 97.19>70.05 (-27.92%) MaxEnt donor site: 10.57>2.92 (-72.37%) | YES |
| **Case 14** | Zhang et al, 2018^12^ | c.1718C>T;p.T573I | Not provided | 0.038 | 0.0001/0/0.0009/0 | No alteration | NO |
|  |  | c.991A>G;p.I331V | Not provided | 0.409 | 0.0142/0/0.0079/0 | No alteration | YES |
| **Case 15** | Xu et al., 2020^13^ | c.773G>A;p.R258H | Not provided | 0.138 | 0.0003/0.0029/0/0 | ESE alteration | YES |
|  |  | c.282_285delAGAG;p.R94Sfs*3 | Not provided | N/A | N/A | Cryptic donor | YES |
| **Case 16** | Minardi et al, 2020^14^ | c.1026G>A;p.M342I | Not provided | 0.061 | 0/0/0/0.01 | No alteration | NO |
|  |  | c.1305+1G>A | Not provided | 0.373 |  | WT donor | YES |
| **Case 17** | Edvarson et al, 2007^15^ | c.110+5A>G, hom | Functionally validated: skipping of exon 2^15^ | 0.716 | 0/0/0.5162/0.7199 | Cryptic donor | YES |
| **Case 18** | Edvarson et al, 2007^15^ | c.110+5A>G, hom | Functionally validated: skipping of exon 2^15^ | 0.716 | 0/0/0.5162/0.7199 | Cryptic donor | YES |
| **Case 19** | Edvarson et al, 2007^15^ | c.110+5A>G, hom | Functionally validated: skipping of exon 2^15^ | 0.716 | 0/0/0.5162/0.7199 | Cryptic donor | YES |
| **Case 20** | Namavar et al, 2011^16^ | c.110+5A>G | Not provided | 0.716 | 0/0/0.5162/0.7199 | Cryptic donor | YES |
|  |  | c.35A>G;p.Q12R | Not provided | 0.968 | 0/0/0.003/0.5824 | ESS creation, ESE alteration | YES |
| **Case 21** | Cassandrini et al, 2013^5^ | c.25G>A;p.I9V | Not provided | 0.138 | 0.0002/0/0/0.0072 | ESS creation | YES |
|  |  | c.1586+3A>T | Functionally validated: splicing mutation deletes 25 amino acids in the anticodon binding domain, shortened but functional version of the protein^5^ | 0.968 | 0.0161/0/0.0079/0.9258 | WT donor: 82.43>69.55 (-15.63%), MaxEnt donor: 8.4>3.87 (-53.93%) | YES |
| **Case 22** | Cassandrini et al, 2013^5^ | c.721T>A;p.W241R | Not provided | 0.681 | 0/0/0.0001/0 | Cryptic acceptor: 48.72>76.59 (57.2%), ESS creation, ESE alteration | YES |
|  |  | c.35A>G;p.Q12R | Functionally validated: insertion of an intron with a premature stop codon^5^ | 0.968 | 0/0/0.003/0.5824 | ESS creation, ESE alteration | YES |
| **Case 23** | Joseph et al, 2014^17^ | c.997C>G;p.R333G | Not provided | 0.001 | 0/0/0/0 | ESS creation, ESE alteration | YES |
|  |  | c.1432G>A;p.G478R | Not provided | 0.442 | 0.0009/0.3341/0/0 | Cryptic acceptor: 45.49>73.36 (61.27%), ESS creation, ESE alteration | YES |
| **Case 24** | Joseph et al, 2014^17^ | c.997C>G;p.R333G | Not provided | 0.001 | 0/0/0/0 | ESS creation, ESE alteration | YES |
|  |  | c.1432G>A;p.G478R | Not provided | 0.442 | 0.0009/0.3341/0/0 | Cryptic acceptor: 45.49>73.36 (61.27%), ESS creation, ESE alteration | YES |
| **Case 25** | Joseph et al, 2014^17^ | c.997C>G;p.R333G | Not provided | 0.001 | 0/0/0/0 | ESS creation, ESE alteration | YES |
|  |  | c.1432G>A;p.G478R | Not provided | 0.442 | 0.0009/0.3341/0/0 | Cryptic acceptor: 45.49>73.36 (61.27%), ESS creation, ESE alteration | YES |
| **Case 26** | Lax et al, 2015^18^ | c.613-3972C>T | Functionally validated: intron retention and skipping of exons 6-8^18^ | 0.679 | 0/00/0.01/0 | HSF cryptic donor:  61.62>88.76 (44.04%) MaxEnt donor:  1.37>9.12 (565.69%) | YES |
|  |  | c.1A>G;p.M1V | Start-loss^18^ | 0 | 0/0/0/0 | Cryptic donor:  37.94>65.08 (71.53%) | YES |
| **Case 27** | Lax et al, 2015^18^ | c.613-3972C>T | Functionally validated: intron retention and skipping of exons 6-8^18^ | 0.679 | N/A | HSF cryptic donor:  61.62>88.76 (44.04%) MaxEnt donor:  1.37>9.12 (565.69%) | YES |
|  |  | c.1A>G;p.M1V | Start-loss^18^ | 0 | 0/0/0/0 | Cryptic donor:  37.94>65.08 (71.53%) | YES |
| **Case 28** | Li et al, 2015^19^ | c.-2A>G, promoter, hom | Functionally validated: reduced promoter activity^19^ | 0.05 | 0.0011/0/0.2857/0.0887 | Not implemented | YES |
| **Case 29** | Li et al, 2015^19^ | c.-2A>G, promoter, hom | Functionally validated: reduced promoter activity^19^ | 0.05 | 0.0011/0/0.2857/0.0887 | Not implemented | YES |
| **Case 30** | Alkhateeb et al, 2016^20^ | c.1588C>T;p.H530Y, hom | Not provided | 0.28 | 0/0/0.0001/0 | No alteration | YES |
| **Case 31** | Alkhateeb et al, 2016^20^ | c.1588C>T;p.H530Y, hom | Not provided | 0.28 | 0/0/0.0001/0 | No alteration | YES |
| **Case 32** | Alkhateeb et al, 2016^20^ | c.1588C>T;p.H530Y, hom | Not provided | 0.28 | 0/0/0.0001/0 | No alteration | YES |
| **Case 33** | Legati et al, 2016^21^ | c.1A>G;p.M1V | Start-loss^21^ | 0 | 0/0/0/0 | Cryptic donor:  37.94>65.08 (71.53%) | YES |
|  |  | p.S443P (c.1327T>C?) | Not provided | 0.015 | 0.0047/0/0/0 | No alteration | NO |
| **Case 34** | Lühl et al, 2016^22^ | c.392T>G;p.F131C, hom | Not provided | 0.104 | 0/0/0.0002/0 | ESS creation, ESE alteration | YES |
| **Case 35** | Lühl et al, 2016^22^ | c.392T>G;p.F131C, hom | Not provided | 0.104 | 0/0/0.0002/0 | ESS creation, ESE alteration | YES |
| **Case 36** | Pronicka et al,  2016^23^ | c.1026G>A;p.M342I | Not provided | 0.061 | 0/0/0/0.01 | No alteration | NO |
|  |  | c.622C>T;p.Q208* | Not provided | 0.066 | 0/0/0/0 | ESE alteration | YES |
| **Case 37** | Van Dijk et al, 2017^11^ | c.1544A>G;p.D515G | Splicing effect considered based on location^11^ | 0.084 | 0.0012/0.1197/0.0007/0 | ESE alteration | YES |
|  |  | c.453_454insC;p.N152Kfs*40 | Possible NMD^11^ | N/A | N/A | No alteration | YES |
| **Case 38** | Frésard et al, 2019^24^ | c.1612delA;p.T538fs | Possible NMD, functionally validated: *RARS2* as an under-expression outlier^24^ | N/A | N/A | ESE alteration | YES |
|  |  | c.419T>G;p.F140C | Functionally validated: *RARS2* as an under-expression outlier^24^ | 0.358 | 0/0/0.0086/0 | Cryptic donor:  43.69>70.83 (62.12%) | YES |
| **Case 39** | Frésard et al, 2019^24^ | c.1612delA;p.T538fs | Possible NMD, functionally validated: *RARS2* as an under-expression outlier^24^ | N/A | N/A | ESE alteration | YES |
|  |  | c.419T>G;p.F140C | Functionally validated: *RARS2* as an under-expression outlier^24^ | 0.358 | 0/0/0.0086/0 | Cryptic donor:  43.69>70.83 (62.12%) | YES |
| **Case 40** | Gieldon et al, 2018^25^ | c.16C>T;p.R6C | Not provided | 0.015 | 0.0006/0/0/0.0145 | ESS creation | YES |
|  |  | c.1544A>G;p.D515G | Not provided | 0.084 | 0.0012/0.1197/0.0007/0 | ESE alteration | YES |
| **Case 41** | Mathew et al, 2018^26^ | c.848T>A;p.L283Q , hom | Not provided | 0.436 | 0/0/0.0001/0.1564 | ESE alteration, cryptic acceptor:  56.96>84.83 (48.93%) | YES |
| **Case 42** | Mathew et al, 2018^26^ | c.848T>A;p.L283Q, hom | Not provided | 0.436 | 0/0/0.0001/0.1564 | ESE alteration, cryptic acceptor:  56.96>84.83 (48.93%) | YES |
| **Case 43** | Al et al, 2020^27^ | c.633_636delAGAA;p. E212Qfs*7 | Possible NMD | N/A | N/A | ESE alteration, acceptor site:  10.25>70.07 (583.61%) | YES |
|  |  | c.1113-21A>C | Functionally validated: Splicing error deletes exon 14^25^ | 0.044 | 0.0019/0.3523/0/0 | No alteration | YES |
| **Case 44** | Shakya et al, 2019^28^ | c.848T>A;p.L283Q, hom | Not provided | 0.436 | 0/0/0.0001/0.1564 | ESE alteration, cryptic acceptor:  56.96>84.83 (48.93%) | YES |
| **Case 45** | Wu et al, 2020^29^ | c.1210A>G;p.M404V | Not provided | 0.374 | 0/0.21/0/0.01 | ESE alteration, cryptic donor | YES |
|  |  | c.622C>T;p.Q208* | Not provided | 0.066 | 0/0/0/0 | ESE alteration | YES |
| **Case 46** | Jiang et al, 2020^30^ | c.1679G>A;p.R560H | Not provided | 0.018 | 0.01/0/0/0 | No alteration | NO |
|  |  | c.17G>A;p.R6H | Not provided | 0.018 | 0/0/0/0 | No alteration | NO |
| **Case 47** | Jiang et al, 2020^30^ | c.760C>T;p.R254W | Not provided | 0.112 | 0/0/0/0 | ESE alteration | YES |
|  |  | c.14T>C;p.F5S | Not provided | 0.011 | 0/0/0/0 | No alteration | NO |
| **Case 48** | Roux et al, 2021^31^ | c.29C>T;p.A10V | Not provided | 0.19 | 0/0/0/0 | ESE alteration, cryptic donor | YES |
|  |  | c.298-1G>A | Not provided | 0.601 | 0.99/0/0.26/0 | WT acceptor | YES |
| **Case 49** | Roux et al, 2021^31^ | c.442A>G;p.T148A | Not provided | 0.007 | 0/0/0.01/0 | ESE alteration | YES |
|  |  | c.472_474del;p.K158delK | Not provided | N/A | N/A | ESE alteration, cryptic acceptor:  58.16>75.4 (29.64%) | YES |
| **Case 50** | Roux et al, 2021^31^ | c.35A>G;p.Q12R | Not provided | 0.968 | 0/0/0.003/0.5824 | ESS creation, ESE alteration | YES |
|  |  | c.601C>G;p.H201D | Not provided | 0.157 | 0/0/0/0.02 | Cryptic acceptor | YES |
| **Case 51** | Roux et al, 2021^31^ | c.965A>G;p.Y322C | Not provided | 0.343 | 0/0.11/0/0 | ESE alteration | YES |
|  |  | c.1564G>A;p.V522I | Not provided | 0.083 | 0/0.01/0/0 | No alteration | NO |
| **Case 52** | Weng et al, 2021^32^ | c.1A>G;p.M1V | Not provided | 0 | 0/0/0/0 | No alteration | YES |
|  |  | c.1564G>A;p.V522I | Not provided | 0.083 | 0/0.01/0/0 | No alteration | NO |

**Supplementary Table 1**. Splicing predictions for variants in our patients and all published *RARS2* cases.

Concordance between *in silico* splicing prediction tools provides the strongest support for aberrant splicing^33^. The +5 position in the donor site is the most highly conserved site after the invariant positions (e.g. +1,+2)^34^ , and one of the most frequently mutated including as shown in our own work on a patient with GLUT1 encephalopathy^35^.

*Variant is present in ClinVar classified as pathogenic, under the accession VCV000974848.1.

We are using the following cut-of values for the predictions:

- TraP score: In coding regions, over 0.221 for the prediction of possibly damaging and over 0.416 for probably damaging. In non-coding regions, 0.174 for possibly damaging and 0.289 for probably damaging^1^.
- SpliceAI: Over 0.2 for ‘high recall’, 0.5 for ‘recommended’ and 0.8 for ‘high precission’^2^.

Abbreviations: AG: “Acceptor gain”; AL: “Acceptor loss”; DG: “Donor gain”; DL ; ESE alteration: “Alteration of an exonic splicing enhancer site. Potential alteration of splicing.”; No alteration: “No significant splicing motif alteration detected. This mutation has probably no impact on splicing.”; WT donor: “Alteration of the wild type donor site, most probably affecting splicing.”; Cryptic donor: “Activation of an intronic cryptic donor site. Potential alteration of splicing.”; Cryptic acceptor: “Activation of an exonic cryptic acceptor site, with presence of one or more cryptic branch point(s).”; ESS creation: “Creation of an exonic splicing silencer site. Potential alteration of splicing.”; Not implemented: “Mutant type not implemented in Human Splicing Finder yet.”; NMD: Nonsense mediated decay; NR: Not recorded; N/A: Not available

**Supplementary Table References**

1. Gelfman S, Wang Q, McSweeney KM, Ren Z, La Carpia F, Halvorsen M, et al. Annotating pathogenic non-coding variants in genic regions. Nat Commun. 2017;8(1):236.

2. Jaganathan K, Kyriazopoulou Panagiotopoulou S, McRae JF, Darbandi SF, Knowles D, Li YI, et al. Predicting Splicing from Primary Sequence with Deep Learning. Cell. 2019;176(3):535–548.e24.

3. Desmet FO, Hamroun D, Lalande M, Collod-Bëroud G, Claustres M, Béroud C. Human Splicing Finder: An online bioinformatics tool to predict splicing signals. Nucleic Acids Res. 2009;37(9):e67.

4. Glamuzina E, Brown R, Hogarth K, Saunders D, Russell-Eggitt I, Pitt M, et al. Further delineation of pontocerebellar hypoplasia type 6 due to mutations in the gene encoding mitochondrial arginyl-tRNA synthetase, *RARS2*. J Inherit Metab Dis. 2012;35(3):459–67.

5. Cassandrini D, Cilio MR, Bianchi M, Doimo M, Balestri M, Tessa A, et al. Pontocerebellar hypoplasia type 6 caused by mutations in *RARS2*: Definition of the clinical spectrum and molecular findings in five patients. J Inherit Metab Dis. 2013;36(1):43–53.

6. Kastrissianakis K, Anand G, Quaghebeur G, Price S, Prabhakar P, Marinova J, et al. Subdural effusions and lack of early pontocerebellar hypoplasia in siblings with *RARS2* mutations. Arch Dis Child. 2013;98(12):1004–7.

7. Rankin J, Brown R, Dobyns WB, Harington J, Patel J, Quinn M, et al. Pontocerebellar hypoplasia type 6: A British case with PEHO-like features. Am J Med Genet Part A. 2010;152(8):2079–84.

8. Nevanlinna V, Konovalova S, Ceulemans B, Muona M, Laari A, Hilander T, et al. A patient with pontocerebellar hypoplasia type 6: Novel *RARS2* mutations, comparison to previously published patients and clinical distinction from PEHO syndrome. Eur J Med Genet. 2020;63(3):103766.

9. Ngoh A, Bras J, Guerreiro R, Meyer E, McTague A, Dawson E, et al. *RARS2* mutations in a sibship with infantile spasms. Epilepsia. 2016;57(5):e97–102.

10. Nishri D, Goldberg-stern H, Noyman I, Blumkin L, Kivity S, Saitsu H. *RARS2* mutations cause early onset epileptic encephalopathy without ponto-cerebellar hypoplasia. Eur J Paediatr Neurol [Internet]. 2016;20(3):412–7. Available from: http://dx.doi.org/10.1016/j.ejpn.2016.02.012

11. van Dijk T, van Ruissen F, Jaeger B, Rodenburg RJ, Tamminga S, van Maarle M, et al. *RARS2* mutations: Is pontocerebellar hypoplasia type 6 a mitochondrial encephalopathy? In: JIMD Reports. 2017;33:87-92.

12. Zhang J, Zhang Z, Zhang Y, Wu Y. Distinct magnetic resonance imaging features in a patient with novel *RARS2* mutations: A case report and review of the literature. Vol. 15, Experimental and Therapeutic Medicine. 2018;15(1):1099–1104.

13. Xu Y, Wu B-B, Wang H-J, Zhou S-Z, Cheng G-Q, Zhou Y-F. A term neonate with early myoclonic encephalopathy caused by *RARS2* gene variants: a case report. Transl Pediatr [Internet]. 2020 Oct;9(5):707–12. Available from: http://tp.amegroups.com/article/view/50764/html

14. Minardi R, Licchetta L, Baroni MC, Pippucci T, Stipa C, Mostacci B, et al. Whole‐exome sequencing in adult patients with developmental and epileptic encephalopathy: It is never too late. Clin Genet [Internet]. 2020 Nov;98(5):477–85. Available from: https://onlinelibrary.wiley.com/doi/10.1111/cge.13823

15. Edvardson S, Shaag A, Kolesnikova O, Gomori JM, Tarassov I, Einbinder T, et al. Deleterious mutation in the mitochondrial arginyl-transfer RNA synthetase gene is associated with pontocerebellar hypoplasia. Am J Hum Genet. 2007;81(4):857–62.

16. Namavar Y, Barth PG, Kasher PR, Van Ruissen F, Brockmann K, Bernert G, et al. Clinical, neuroradiological and genetic findings in pontocerebellar hypoplasia. Brain. 2011;134(1):143–56.

17. Joseph JT, Innes AM, Smith AC, Vanstone MR, Schwartzentruber JA, Consortium FC, et al. Neuropathologic Features of Pontocerebellar Hypoplasia Type 6. 2014;73(11):1009–25.

18. Lax NZ, Alston CL, Schon K, Park SM, Krishnakumar D, He L, et al. Neuropathologic Characterization of Pontocerebellar Hypoplasia Type 6 Associated With Cardiomyopathy and Hydrops Fetalis and Severe Multisystem Respiratory Chain Deficiency due to Novel *RARS2* Mutations. J Neuropathol Exp Neurol. 2015;74(7):688–703.

19. Li Z, Schonberg R, Guidugli L, Johnson AK, Arnovitz S, Yang S, et al. A novel mutation in the promoter of *RARS2* causes pontocerebellar hypoplasia in two siblings. J Hum Genet. 2015;60(7):363–9.

20. Alkhateeb AM, Aburahma SK, Habbab W, Thompson IR. Novel mutations in *WWOX* , *RARS2* , and C10orf2 genes in consanguineous Arab families with intellectual disability. Metab Brain Dis [Internet]. 2016;31(4):901–907. Available from: http://dx.doi.org/10.1007/s11011-016-9827-9

21. Legati A, Reyes A, Nasca A, Invernizzi F, Lamantea E, Tiranti V, et al. New genes and pathomechanisms in mitochondrial disorders unraveled by NGS technologies. Biochim Biophys Acta - Bioenerg. 2016;1857(8):1326–35.

22. Lühl S, Bode H, Schlötzer W, Bartsakoulia M, Horvath R, Abicht A, et al. Novel homozygous *RARS2* mutation in two siblings without pontocerebellar hypoplasia – further expansion of the phenotypic spectrum. 2016;11(1):;140.

23. Pronicka E, Abramczuk DP, Ciara E, Trubicka J, Rokicki D, Więckowska AK, et al. New perspective in diagnostics of mitochondrial disorders : two years ’ experience with whole ‑ exome sequencing at a national paediatric centre. J Transl Med. 2016; 14(1):174.

24. Frésard L, Smail C, Ferraro NM, Teran NA, Li X, Smith KS, et al. Identification of rare-disease genes using blood transcriptome sequencing and large control cohorts. Vol. 25, Nature Medicine. 2019; 25(6):911–919.

25. Gieldon L, Mackenroth L, Kahlert A, Lemke JR, Porrmann J, Schallner J, et al. Diagnostic value of partial exome sequencing in developmental disorders. 2018;13(8):1–16.

26. Mathew T, Avati A, D’Souza D, Therambil M. Expanding spectrum of *RARS2* gene disorders: Myoclonic epilepsy, mental retardation, spasticity, and extrapyramidal features. Epilepsia Open. 2018;3(2):270–5.

27. Al A, Diana B, Rebekah M, Salomons GS, Mercimek-andrews SBS. Phenotypes and genotypes of mitochondrial aminoacyl-tRNA synthetase deficiencies from a single neurometabolic clinic. 2020;2(July 2019):3–10.

28. Shakya S, Kumari R, Suroliya V, Tyagi N, Joshi A, Garg A, et al. Whole exome and targeted gene sequencing to detect pathogenic recessive variants in early onset cerebellar ataxia. Clin Genet. 2019;96(6):566–74.

29. Wu TH, Peng J, Zhang CL, Wu LW, Yang LF, Peng P, et al. Mutations in aminoacyl-tRNA synthetase genes: An analysis of 10 cases. Chinese J Contemp Pediatr. 2020; 22(6):595-601

30. Jiang HF, Deng J, Fang F, Li H, Wang XH, Dai LF. [Early onset epileptic encephalopathy caused by mitochondrial arginyl-tRNA synthetase gene deficiency: report of two cases and literature review]. Zhonghua er ke za zhi = Chinese J Pediatr [Internet]. 2020 Nov 2;58(11):893–9. Available from: http://www.ncbi.nlm.nih.gov/pubmed/33120460

31. Roux C-J, Barcia G, Schiff M, Sissler M, Levy R, Dangouloff-Ros V, et al. Phenotypic diversity of brain MRI patterns in mitochondrial aminoacyl-tRNA synthetase mutations. Mol Genet Metab [Internet]. 2021;133(2):222–9. Available from: http://www.ncbi.nlm.nih.gov/pubmed/33972171

32. Weng X, Liu Y, Peng Y, Liang Z, Jin X, Cheng L, et al. [Analysis of genetic variant in a fetus featuring pontocerebellar hypoplasia type 6]. Zhonghua Yi Xue Yi Chuan Xue Za Zhi [Internet]. 2021 Jul 10;38(7):667–70. Available from: http://www.ncbi.nlm.nih.gov/pubmed/34247374

33. Baert A, Machackova E, Coene I, Cremin C, Turner K, Portigal-Todd C, et al. Thorough in silico and in vitro cDNA analysis of 21 putative BRCA1 and BRCA2 splice variants and a complex tandem duplication in BRCA2 allowing the identification of activated cryptic splice donor sites in BRCA2 exon 11. Hum Mutat. 2018;39(4):515–26.

34. Shapiro MB, Senapathy P. RNA splice junctions of different classes of eukaryotes: Sequence statistics and functional implications in gene expression. Nucleic Acids Res. 1987;15(17):7155–74.

35. Liu YC, Lee JWA, Bellows ST, Damiano JA, Mullen SA, Berkovic SF, et al. Evaluation of non-coding variation in GLUT1 deficiency. Dev Med Child Neurol. 2016;58(12):1295–302.
