# Supplementary material for: Infantile‐onset myoclonic developmental and epileptic encephalopathy: A new RARS2 phenotype
Source: Epilepsia Open. 2021 Nov 18;7(1):170–80. doi: 10.1002/epi4.12553 (PMC8886097; doi:10.1002/epi4.12553)
Supplement: Supplementary file 2 — Table S2 [file EPI4-7-170-s001.docx]

| Publication | Ars et al., 2000^1^ | | | Maruoka et al., 2014^2^ | | Zafar et al., 2016^3^ |
| --- | --- | --- | --- | --- | --- | --- |
| Patient | 92-152 | 96-216 (mother) | 96-216  (daughter) | Exon 18: 23 y | Exon 18: 35 y | Case 1 |
| Epilepsy | NO | NO | NO | NO | NO | NO |
| NF1 manifestation | CaL, cutaneous neurofibromas | CaL, cutaneous neurofibromas | CaL, cutaneous neurofibromas | CaL, cutaneous neurofibromas | CaL, cutaneous neurofibromas | CaL, right axillary freckling |
| Other findings | ID | None | Scoliosis, Lisch nodules | None | Bone manifestations | Fragile X; At birth: hypotonia, lethargic, poor feeding; Profound DD; Right optic nerve glioma, hypothalamic glioma and brainstem glioma. |

**Supplementary Table 2**. Published patients with the same *NF1* variant as Case B (*NF1* p.R681X). Abbreviations: CaL, café-au-lait spots; DD, development delay; ID, intellectual disability; y, years.

**Supplementary Table 2 Bibliography:**

1. Ars E, Serra E, García J, Kruyer H, Gaona A, Lázaro C, et al. Mutations affecting mRNA splicing are the most common molecular defects in patients with neurofibromatosis type 1. Hum Mol Genet. 2000;

2. Maruoka R, Takenouchi T, Torii C, Shimizu A, Misu K, Higasa K, et al. The use of next-generation sequencing in molecular diagnosis of neurofibromatosis type 1: a validation study. Genet Test Mol Biomarkers. 2014;

3. Zafar R, Hsiao EY, Botteron KN, McKinstry RC, Gutmann DH. De novo development of gliomas in a child with neurofibromatosis type 1, fragile X and previously normal brain magnetic resonance imaging. Radiol Case Reports. 2016;
